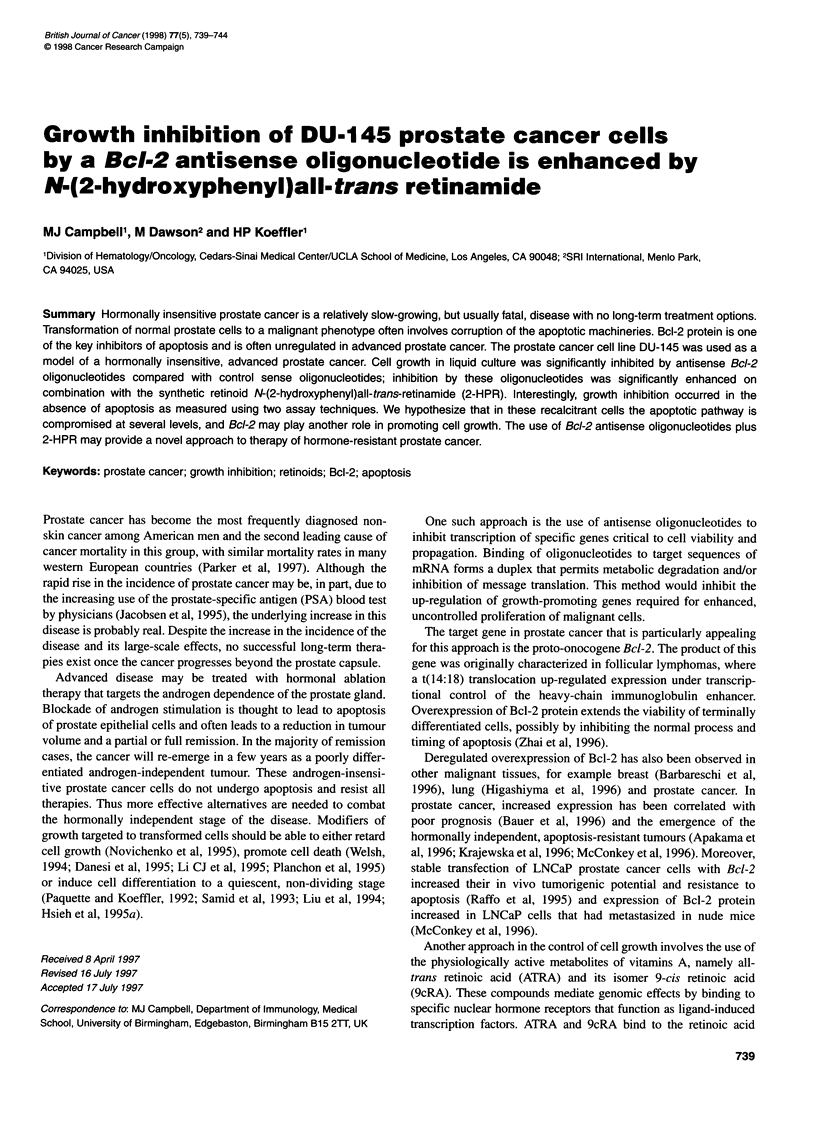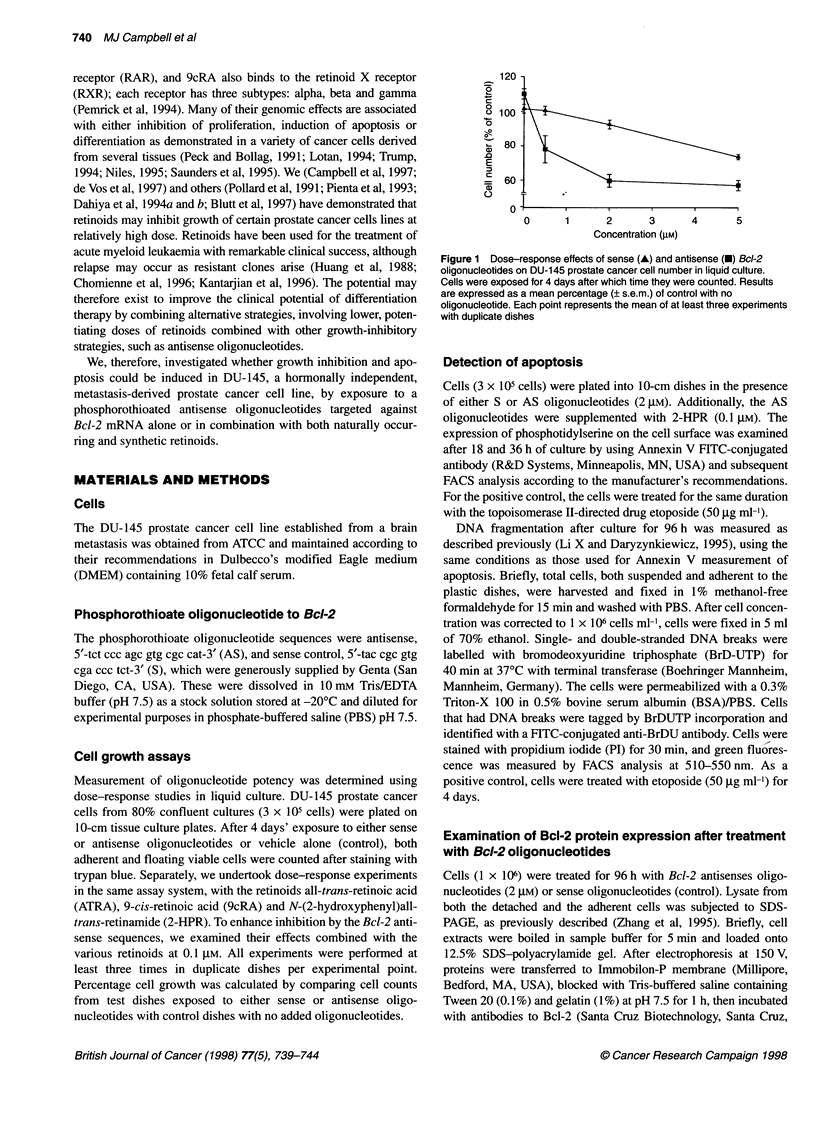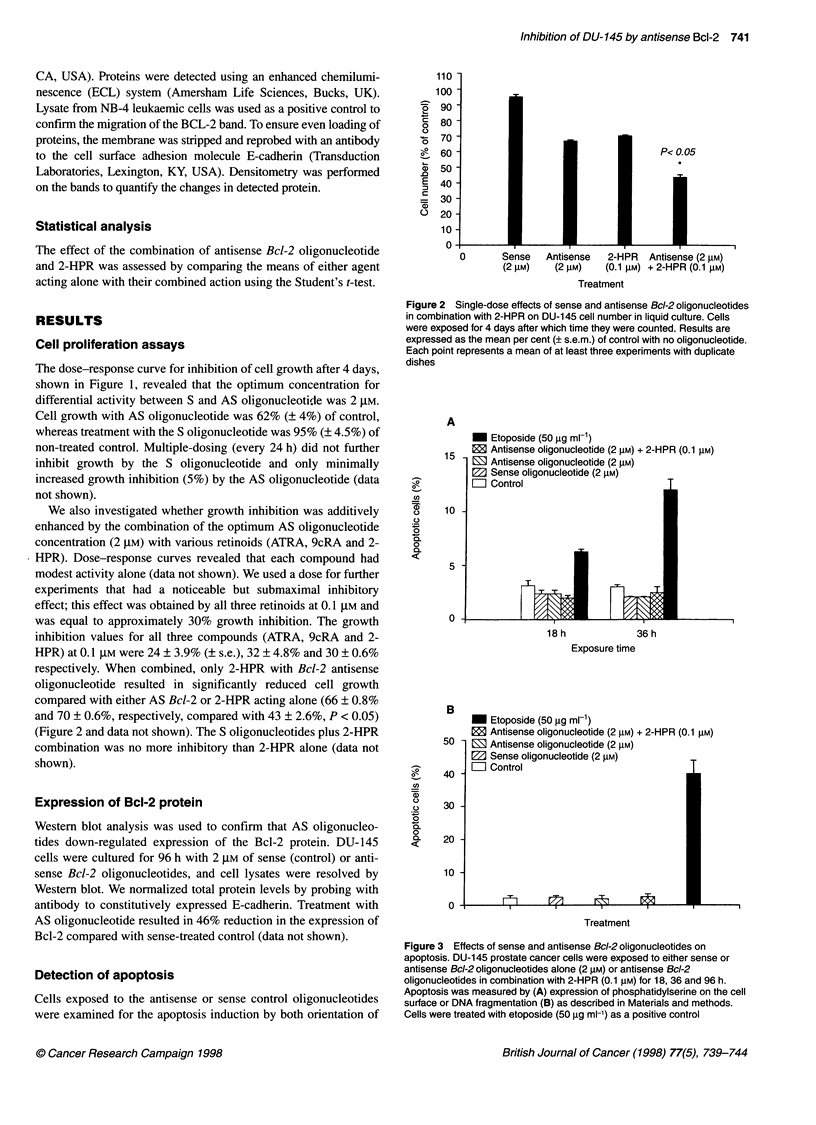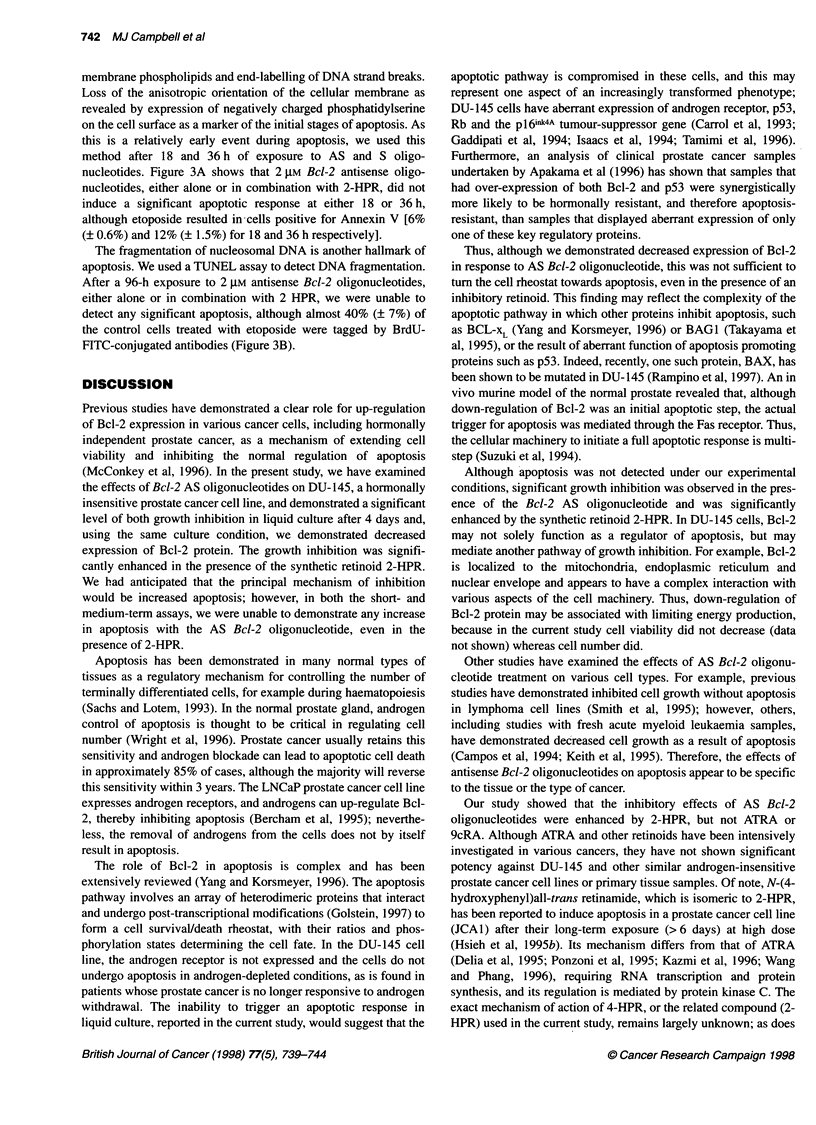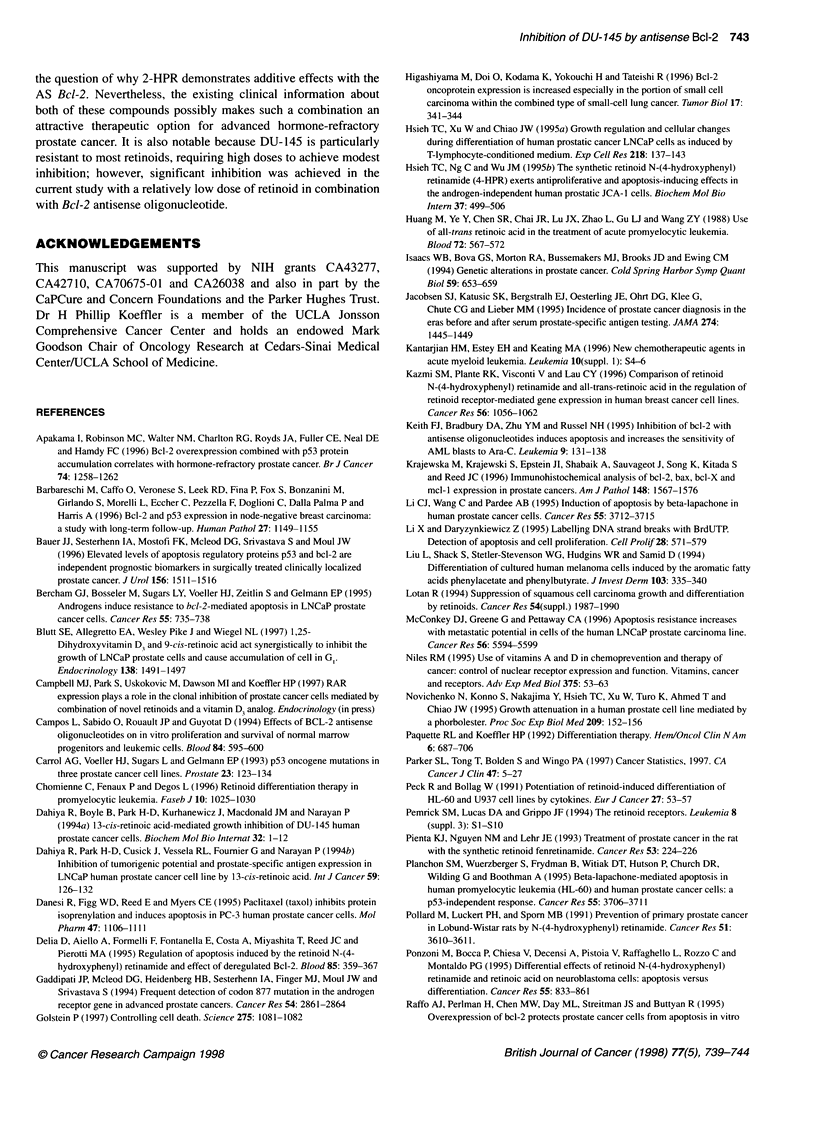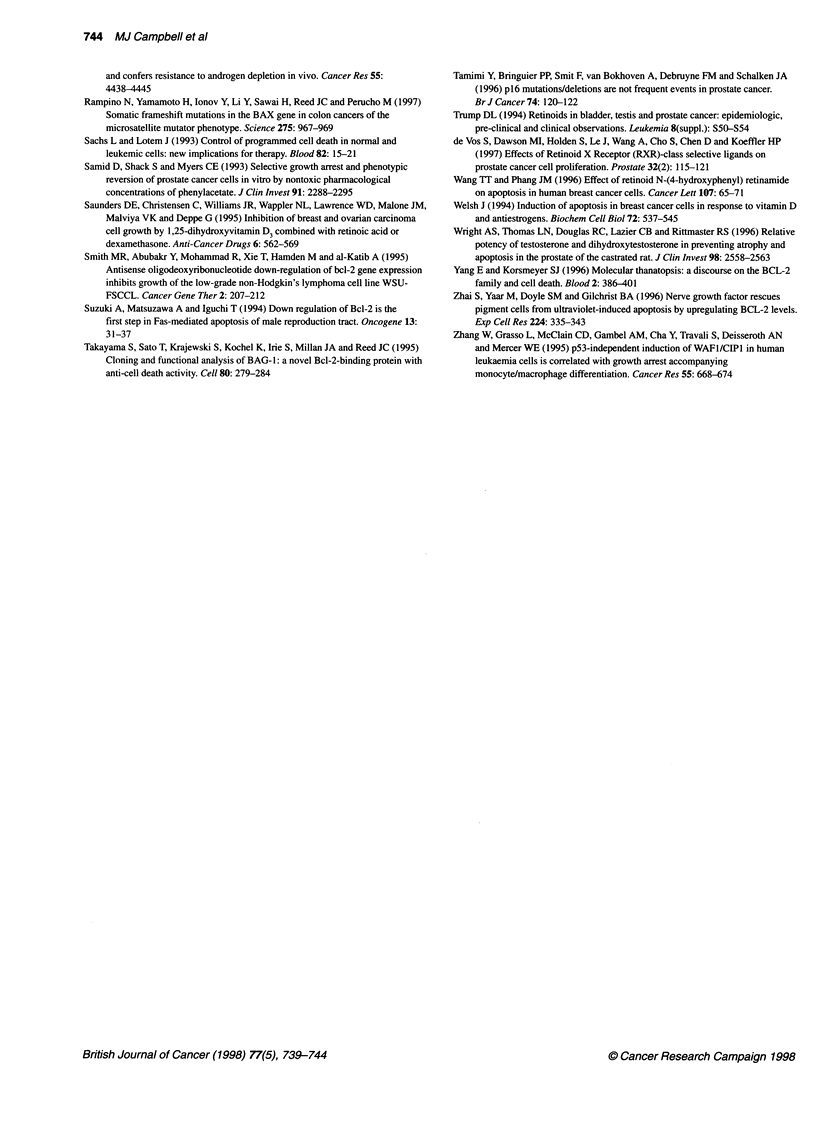# Growth inhibition of DU-145 prostate cancer cells by a Bcl-2 antisense oligonucleotide is enhanced by N-(2-hydroxyphenyl)all-trans retinamide.

**DOI:** 10.1038/bjc.1998.121

**Published:** 1998-03

**Authors:** M. J. Campbell, M. Dawson, H. P. Koeffler

**Affiliations:** Division of Hematology/Oncology, Cedars-Sinai Medical Center/UCLA School of Medicine, Los Angeles, CA 90048, USA.

## Abstract

Hormonally insensitive prostate cancer is a relatively slow-growing, but usually fatal, disease with no long-term treatment options. Transformation of normal prostate cells to a malignant phenotype often involves corruption of the apoptotic machineries. Bcl-2 protein is one of the key inhibitors of apoptosis and is often unregulated in advanced prostate cancer. The prostate cancer cell line DU-145 was used as a model of a hormonally insensitive, advanced prostate cancer. Cell growth in liquid culture was significantly inhibited by antisense Bcl-2 oligonucleotides compared with control sense oligonucleotides; inhibition by these oligonucleotides was significantly enhanced on combination with the synthetic retinoid N-(2-hydroxyphenyl)all-trans-retinamide (2-HPR). Interestingly, growth inhibition occurred in the absence of apoptosis as measured using two assay techniques. We hypothesize that in these recalcitrant cells the apoptotic pathway is compromised at several levels, and Bcl-2 may play another role in promoting cell growth. The use of Bcl-2 antisense oligonucleotides plus 2-HPR may provide a novel approach to therapy of hormone-resistant prostate cancer.


British Journal of Cancer (1998) 77(5), 739–744
© 1998 Cancer Research Campaign

### Growth inhibition of DU-145 prostate cancer cells by a *Bcl-2* antisense oligonucleotide is enhanced by *N*-(2-hydroxyphenyl)all-*trans* retinamide

**MJ Campbell[1], M Dawson[2] and HP Koeffler[1]**

[1]Division of Hematology/Oncology, Cedars-Sinai Medical Center/UCLA School of Medicine, Los Angeles, CA 90048; [2]SRI International, Menlo Park, CA 94025, USA

**Summary** Hormonally insensitive prostate cancer is a relatively slow-growing, but usually fatal, disease with no long-term treatment options. Transformation of normal prostate cells to a malignant phenotype often involves corruption of the apoptotic machineries. Bcl-2 protein is one of the key inhibitors of apoptosis and is often unregulated in advanced prostate cancer. The prostate cancer cell line DU-145 was used as a model of a hormonally insensitive, advanced prostate cancer. Cell growth in liquid culture was significantly inhibited by antisense *Bcl-2* oligonucleotides compared with control sense oligonucleotides; inhibition by these oligonucleotides was significantly enhanced on combination with the synthetic retinoid *N*-(2-hydroxyphenyl)all-*trans*-retinamide (2-HPR). Interestingly, growth inhibition occurred in the absence of apoptosis as measured using two assay techniques. We hypothesize that in these recalcitrant cells the apoptotic pathway is compromised at several levels, and *Bcl-2* may play another role in promoting cell growth. The use of *Bcl-2* antisense oligonucleotides plus 2-HPR may provide a novel approach to therapy of hormone-resistant prostate cancer.

**Keywords:** prostate cancer; growth inhibition; retinoids; Bcl-2; apoptosis

Prostate cancer has become the most frequently diagnosed non-skin cancer among American men and the second leading cause of cancer mortality in this group, with similar mortality rates in many western European countries (Parker et al, 1997). Although the rapid rise in the incidence of prostate cancer may be, in part, due to the increasing use of the prostate-specific antigen (PSA) blood test by physicians (Jacobsen et al, 1995), the underlying increase in this disease is probably real. Despite the increase in the incidence of the disease and its large-scale effects, no successful long-term therapies exist once the cancer progresses beyond the prostate capsule.

Advanced disease may be treated with hormonal ablation therapy that targets the androgen dependence of the prostate gland. Blockade of androgen stimulation is thought to lead to apoptosis of prostate epithelial cells and often leads to a reduction in tumour volume and a partial or full remission. In the majority of remission cases, the cancer will re-emerge in a few years as a poorly differentiated androgen-independent tumour. These androgen-insensitive prostate cancer cells do not undergo apoptosis and resist all therapies. Thus more effective alternatives are needed to combat the hormonally independent stage of the disease. Modifiers of growth targeted to transformed cells should be able to either retard cell growth (Novichenko et al, 1995), promote cell death (Welsh, 1994; Danesi et al, 1995; Li CJ et al, 1995; Planchon et al, 1995) or induce cell differentiation to a quiescent, non-dividing stage (Paquette and Koeffler, 1992; Samid et al, 1993; Liu et al, 1994; Hsieh et al, 1995*a*).

*Received 8 April 1997*
*Revised 16 July 1997*
*Accepted 17 July 1997*

*Correspondence to*: MJ Campbell, Department of Immunology, Medical School, University of Birmingham, Edgebaston, Birmingham B15 2TT, UK

One such approach is the use of antisense oligonucleotides to inhibit transcription of specific genes critical to cell viability and propagation. Binding of oligonucleotides to target sequences of mRNA forms a duplex that permits metabolic degradation and/or inhibition of message translation. This method would inhibit the up-regulation of growth-promoting genes required for enhanced, uncontrolled proliferation of malignant cells.

The target gene in prostate cancer that is particularly appealing for this approach is the proto-onocogene *Bcl-2*. The product of this gene was originally characterized in follicular lymphomas, where a t(14:18) translocation up-regulated expression under transcriptional control of the heavy-chain immunoglobulin enhancer. Overexpression of Bcl-2 protein extends the viability of terminally differentiated cells, possibly by inhibiting the normal process and timing of apoptosis (Zhai et al, 1996).

Deregulated overexpression of Bcl-2 has also been observed in other malignant tissues, for example breast (Barbareschi et al, 1996), lung (Higashiyma et al, 1996) and prostate cancer. In prostate cancer, increased expression has been correlated with poor prognosis (Bauer et al, 1996) and the emergence of the hormonally independent, apoptosis-resistant tumours (Apakama et al, 1996; Krajewska et al, 1996; McConkey et al, 1996). Moreover, stable transfection of LNCaP prostate cancer cells with *Bcl-2* increased their in vivo tumorigenic potential and resistance to apoptosis (Raffo et al, 1995) and expression of Bcl-2 protein increased in LNCaP cells that had metastasized in nude mice (McConkey et al, 1996).

Another approach in the control of cell growth involves the use of the physiologically active metabolites of vitamins A, namely all-*trans* retinoic acid (ATRA) and its isomer 9-*cis* retinoic acid (9cRA). These compounds mediate genomic effects by binding to specific nuclear hormone receptors that function as ligand-induced transcription factors. ATRA and 9cRA bind to the retinoic acid receptor (RAR), and 9cRA also binds to the retinoid X receptor (RXR); each receptor has three subtypes: alpha, beta and gamma (Pemrick et al, 1994). Many of their genomic effects are associated with either inhibition of proliferation, induction of apoptosis or differentiation as demonstrated in a variety of cancer cells derived from several tissues (Peck and Bollag, 1991; Lotan, 1994; Trump, 1994; Niles, 1995; Saunders et al, 1995). We (Campbell et al, 1997; de Vos et al, 1997) and others (Pollard et al, 1991; Pienta et al, 1993; Dahiya et al, 1994*a* and *b*; Blutt et al, 1997) have demonstrated that retinoids may inhibit growth of certain prostate cancer cells lines at relatively high dose. Retinoids have been used for the treatment of acute myeloid leukaemia with remarkable clinical success, although relapse may occur as resistant clones arise (Huang et al, 1988; Chomienne et al, 1996; Kantarjian et al, 1996). The potential may therefore exist to improve the clinical potential of differentiation therapy by combining alternative strategies, involving lower, potentiating doses of retinoids combined with other growth-inhibitory strategies, such as antisense oligonucleotides.

We, therefore, investigated whether growth inhibition and apoptosis could be induced in DU-145, a hormonally independent, metastasis-derived prostate cancer cell line, by exposure to a phosphorothioated antisense oligonucleotides targeted against *Bcl-2* mRNA alone or in combination with both naturally occurring and synthetic retinoids.

### MATERIALS AND METHODS

### Cells

The DU-145 prostate cancer cell line established from a brain metastasis was obtained from ATCC and maintained according to their recommendations in Dulbecco's modified Eagle medium (DMEM) containing 10% fetal calf serum.

### Phosphorothioate oligonucleotide to *Bcl-2*

The phosphorothioate oligonucleotide sequences were antisense, 5′-tct ccc agc gtg cgc cat-3′ (AS), and sense control, 5′-tac cgc gtg cga ccc tct-3′ (S), which were generously supplied by Genta (San Diego, CA, USA). These were dissolved in 10 mM Tris/EDTA buffer (pH 7.5) as a stock solution stored at –20°C and diluted for experimental purposes in phosphate-buffered saline (PBS) pH 7.5.

### Cell growth assays

Measurement of oligonucleotide potency was determined using dose–response studies in liquid culture. DU-145 prostate cancer cells from 80% confluent cultures (3 × $10^5$ cells) were plated on 10-cm tissue culture plates. After 4 days' exposure to either sense or antisense oligonucleotides or vehicle alone (control), both adherent and floating viable cells were counted after staining with trypan blue. Separately, we undertook dose–response experiments in the same assay system, with the retinoids all-*trans*-retinoic acid (ATRA), 9-*cis*-retinoic acid (9cRA) and *N*-(2-hydroxyphenyl)all-*trans*-retinamide (2-HPR). To enhance inhibition by the *Bcl-2* antisense sequences, we examined their effects combined with the various retinoids at 0.1 μM. All experiments were performed at least three times in duplicate dishes per experimental point. Percentage cell growth was calculated by comparing cell counts from test dishes exposed to either sense or antisense oligonucleotides with control dishes with no added oligonucleotides.

**Figure 1** Dose–response effects of sense (▲) and antisense (■) *Bcl-2* oligonucleotides on DU-145 prostate cancer cell number in liquid culture. Cells were exposed for 4 days after which time they were counted. Results are expressed as a mean percentage (± s.e.m.) of control with no oligonucleotide. Each point represents the mean of at least three experiments with duplicate dishes

### Detection of apoptosis

Cells (3 × $10^5$ cells) were plated into 10-cm dishes in the presence of either S or AS oligonucleotides (2 μM). Additionally, the AS oligonucleotides were supplemented with 2-HPR (0.1 μM). The expression of phosphotidylserine on the cell surface was examined after 18 and 36 h of culture by using Annexin V FITC-conjugated antibody (R&D Systems, Minneapolis, MN, USA) and subsequent FACS analysis according to the manufacturer's recommendations. For the positive control, the cells were treated for the same duration with the topoisomerase II-directed drug etoposide (50 μg $ml^{-1}$).

DNA fragmentation after culture for 96 h was measured as described previously (Li X and Daryzynkiewicz, 1995), using the same conditions as those used for Annexin V measurement of apoptosis. Briefly, total cells, both suspended and adherent to the plastic dishes, were harvested and fixed in 1% methanol-free formaldehyde for 15 min and washed with PBS. After cell concentration was corrected to 1 × $10^6$ cells $ml^{-1}$, cells were fixed in 5 ml of 70% ethanol. Single- and double-stranded DNA breaks were labelled with bromodeoxyuridine triphosphate (BrD-UTP) for 40 min at 37°C with terminal transferase (Boehringer Mannheim, Mannheim, Germany). The cells were permeabilized with a 0.3% Triton-X 100 in 0.5% bovine serum albumin (BSA)/PBS. Cells that had DNA breaks were tagged by BrDUTP incorporation and identified with a FITC-conjugated anti-BrDU antibody. Cells were stained with propidium iodide (PI) for 30 min, and green fluorescence was measured by FACS analysis at 510–550 nm. As a positive control, cells were treated with etoposide (50 μg $ml^{-1}$) for 4 days.

### Examination of Bcl-2 protein expression after treatment with *Bcl-2* oligonucleotides

Cells (1 × $10^6$) were treated for 96 h with *Bcl-2* antisenses oligonucleotides (2 μM) or sense oligonucleotides (control). Lysate from both the detached and the adherent cells was subjected to SDS-PAGE, as previously described (Zhang et al, 1995). Briefly, cell extracts were boiled in sample buffer for 5 min and loaded onto 12.5% SDS-polyacrylamide gel. After electrophoresis at 150 V, proteins were transferred to Immobilon-P membrane (Millipore, Bedford, MA, USA), blocked with Tris-buffered saline containing Tween 20 (0.1%) and gelatin (1%) at pH 7.5 for 1 h, then incubated with antibodies to Bcl-2 (Santa Cruz Biotechnology, Santa Cruz,

© Cancer Research Campaign 1998

CA, USA). Proteins were detected using an enhanced chemiluminescence (ECL) system (Amersham Life Sciences, Bucks, UK). Lysate from NB-4 leukaemic cells was used as a positive control to confirm the migration of the BCL-2 band. To ensure even loading of proteins, the membrane was stripped and reprobed with an antibody to the cell surface adhesion molecule E-cadherin (Transduction Laboratories, Lexington, KY, USA). Densitometry was performed on the bands to quantify the changes in detected protein.

### Statistical analysis

The effect of the combination of antisense *Bcl-2* oligonucleotide and 2-HPR was assessed by comparing the means of either agent acting alone with their combined action using the Student's *t*-test.

### RESULTS

### Cell proliferation assays

The dose–response curve for inhibition of cell growth after 4 days, shown in Figure 1, revealed that the optimum concentration for differential activity between S and AS oligonucleotide was 2 μM. Cell growth with AS oligonucleotide was 62% (± 4%) of control, whereas treatment with the S oligonucleotide was 95% (± 4.5%) of non-treated control. Multiple-dosing (every 24 h) did not further inhibit growth by the S oligonucleotide and only minimally increased growth inhibition (5%) by the AS oligonucleotide (data not shown).

We also investigated whether growth inhibition was additively enhanced by the combination of the optimum AS oligonucleotide concentration (2 μM) with various retinoids (ATRA, 9cRA and 2-HPR). Dose–response curves revealed that each compound had modest activity alone (data not shown). We used a dose for further experiments that had a noticeable but submaximal inhibitory effect; this effect was obtained by all three retinoids at 0.1 μM and was equal to approximately 30% growth inhibition. The growth inhibition values for all three compounds (ATRA, 9cRA and 2-HPR) at 0.1 μM were 24 ± 3.9% (± s.e.), 32 ± 4.8% and 30 ± 0.6% respectively. When combined, only 2-HPR with *Bcl-2* antisense oligonucleotide resulted in significantly reduced cell growth compared with either AS *Bcl*-2 or 2-HPR acting alone (66 ± 0.8% and 70 ± 0.6%, respectively, compared with 43 ± 2.6%, $P < 0.05$) (Figure 2 and data not shown). The S oligonucleotides plus 2-HPR combination was no more inhibitory than 2-HPR alone (data not shown).

### Expression of Bcl-2 protein

Western blot analysis was used to confirm that AS oligonucleotides down-regulated expression of the Bcl-2 protein. DU-145 cells were cultured for 96 h with 2 μM of sense (control) or antisense *Bcl-2* oligonucleotides, and cell lysates were resolved by Western blot. We normalized total protein levels by probing with antibody to constitutively expressed E-cadherin. Treatment with AS oligonucleotide resulted in 46% reduction in the expression of Bcl-2 compared with sense-treated control (data not shown).

### Detection of apoptosis

Cells exposed to the antisense or sense control oligonucleotides were examined for the apoptosis induction by both orientation of

**Figure 2** Single-dose effects of sense and antisense *Bcl-2* oligonucleotides in combination with 2-HPR on DU-145 cell number in liquid culture. Cells were exposed for 4 days after which time they were counted. Results are expressed as the mean per cent (± s.e.m.) of control with no oligonucleotide. Each point represents a mean of at least three experiments with duplicate dishes

**Figure 3** Effects of sense and antisense *Bcl-2* oligonucleotides on apoptosis. DU-145 prostate cancer cells were exposed to either sense or antisense *Bcl-2* oligonucleotides alone (2 μM) or antisense *Bcl-2* oligonucleotides in combination with 2-HPR (0.1 μM) for 18, 36 and 96 h. Apoptosis was measured by (**A**) expression of phosphatidylserine on the cell surface or DNA fragmentation (**B**) as described in Materials and methods. Cells were treated with etoposide (50 μg ml−1) as a positive control

© Cancer Research Campaign 1998

membrane phospholipids and end-labelling of DNA strand breaks. Loss of the anisotropic orientation of the cellular membrane as revealed by expression of negatively charged phosphatidylserine on the cell surface as a marker of the initial stages of apoptosis. As this is a relatively early event during apoptosis, we used this method after 18 and 36 h of exposure to AS and S oligonucleotides. Figure 3A shows that 2 μM *Bcl-2* antisense oligonucleotides, either alone or in combination with 2-HPR, did not induce a significant apoptotic response at either 18 or 36 h, although etoposide resulted in cells positive for Annexin V [6% (± 0.6%) and 12% (± 1.5%) for 18 and 36 h respectively].

The fragmentation of nucleosomal DNA is another hallmark of apoptosis. We used a TUNEL assay to detect DNA fragmentation. After a 96-h exposure to 2 μM antisense *Bcl-2* oligonucleotides, either alone or in combination with 2 HPR, we were unable to detect any significant apoptosis, although almost 40% (± 7%) of the control cells treated with etoposide were tagged by BrdU-FITC-conjugated antibodies (Figure 3B).

### DISCUSSION

Previous studies have demonstrated a clear role for up-regulation of Bcl-2 expression in various cancer cells, including hormonally independent prostate cancer, as a mechanism of extending cell viability and inhibiting the normal regulation of apoptosis (McConkey et al, 1996). In the present study, we have examined the effects of *Bcl-2* AS oligonucleotides on DU-145, a hormonally insensitive prostate cancer cell line, and demonstrated a significant level of both growth inhibition in liquid culture after 4 days and, using the same culture condition, we demonstrated decreased expression of Bcl-2 protein. The growth inhibition was significantly enhanced in the presence of the synthetic retinoid 2-HPR. We had anticipated that the principal mechanism of inhibition would be increased apoptosis; however, in both the short- and medium-term assays, we were unable to demonstrate any increase in apoptosis with the AS *Bcl-2* oligonucleotide, even in the presence of 2-HPR.

Apoptosis has been demonstrated in many normal types of tissues as a regulatory mechanism for controlling the number of terminally differentiated cells, for example during haematopoiesis (Sachs and Lotem, 1993). In the normal prostate gland, androgen control of apoptosis is thought to be critical in regulating cell number (Wright et al, 1996). Prostate cancer usually retains this sensitivity and androgen blockade can lead to apoptotic cell death in approximately 85% of cases, although the majority will reverse this sensitivity within 3 years. The LNCaP prostate cancer cell line expresses androgen receptors, and androgens can up-regulate Bcl-2, thereby inhibiting apoptosis (Bercham et al, 1995); nevertheless, the removal of androgens from the cells does not by itself result in apoptosis.

The role of Bcl-2 in apoptosis is complex and has been extensively reviewed (Yang and Korsmeyer, 1996). The apoptosis pathway involves an array of heterodimeric proteins that interact and undergo post-transcriptional modifications (Golstein, 1997) to form a cell survival/death rheostat, with their ratios and phosphorylation states determining the cell fate. In the DU-145 cell line, the androgen receptor is not expressed and the cells do not undergo apoptosis in androgen-depleted conditions, as is found in patients whose prostate cancer is no longer responsive to androgen withdrawal. The inability to trigger an apoptotic response in liquid culture, reported in the current study, would suggest that the apoptotic pathway is compromised in these cells, and this may represent one aspect of an increasingly transformed phenotype; DU-145 cells have aberrant expression of androgen receptor, p53, Rb and the p16$^{ink4A}$ tumour-suppressor gene (Carrol et al, 1993; Gaddipati et al, 1994; Isaacs et al, 1994; Tamimi et al, 1996). Furthermore, an analysis of clinical prostate cancer samples undertaken by Apakama et al (1996) has shown that samples that had over-expression of both Bcl-2 and p53 were synergistically more likely to be hormonally resistant, and therefore apoptosis-resistant, than samples that displayed aberrant expression of only one of these key regulatory proteins.

Thus, although we demonstrated decreased expression of Bcl-2 in response to AS *Bcl-2* oligonucleotide, this was not sufficient to turn the cell rheostat towards apoptosis, even in the presence of an inhibitory retinoid. This finding may reflect the complexity of the apoptotic pathway in which other proteins inhibit apoptosis, such as BCL-$x_L$ (Yang and Korsmeyer, 1996) or BAG1 (Takayama et al, 1995), or the result of aberrant function of apoptosis promoting proteins such as p53. Indeed, recently, one such protein, BAX, has been shown to be mutated in DU-145 (Rampino et al, 1997). An in vivo murine model of the normal prostate revealed that, although down-regulation of Bcl-2 was an initial apoptotic step, the actual trigger for apoptosis was mediated through the Fas receptor. Thus, the cellular machinery to initiate a full apoptotic response is multistep (Suzuki et al, 1994).

Although apoptosis was not detected under our experimental conditions, significant growth inhibition was observed in the presence of the *Bcl-2* AS oligonucleotide and was significantly enhanced by the synthetic retinoid 2-HPR. In DU-145 cells, Bcl-2 may not solely function as a regulator of apoptosis, but may mediate another pathway of growth inhibition. For example, Bcl-2 is localized to the mitochondria, endoplasmic reticulum and nuclear envelope and appears to have a complex interaction with various aspects of the cell machinery. Thus, down-regulation of Bcl-2 protein may be associated with limiting energy production, because in the current study cell viability did not decrease (data not shown) whereas cell number did.

Other studies have examined the effects of AS *Bcl-2* oligonucleotide treatment on various cell types. For example, previous studies have demonstrated inhibited cell growth without apoptosis in lymphoma cell lines (Smith et al, 1995); however, others, including studies with fresh acute myeloid leukaemia samples, have demonstrated decreased cell growth as a result of apoptosis (Campos et al, 1994; Keith et al, 1995). Therefore, the effects of antisense *Bcl-2* oligonucleotides on apoptosis appear to be specific to the tissue or the type of cancer.

Our study showed that the inhibitory effects of AS *Bcl-2* oligonucleotides were enhanced by 2-HPR, but not ATRA or 9cRA. Although ATRA and other retinoids have been intensively investigated in various cancers, they have not shown significant potency against DU-145 and other similar androgen-insensitive prostate cancer cell lines or primary tissue samples. Of note, *N*-(4-hydroxyphenyl)all-*trans* retinamide, which is isomeric to 2-HPR, has been reported to induce apoptosis in a prostate cancer cell line (JCA1) after their long-term exposure (> 6 days) at high dose (Hsieh et al, 1995*b*). Its mechanism differs from that of ATRA (Delia et al, 1995; Ponzoni et al, 1995; Kazmi et al, 1996; Wang and Phang, 1996), requiring RNA transcription and protein synthesis, and its regulation is mediated by protein kinase C. The exact mechanism of action of 4-HPR, or the related compound (2-HPR) used in the current study, remains largely unknown; as does

 © Cancer Research Campaign 1998

the question of why 2-HPR demonstrates additive effects with the AS *Bcl-2*. Nevertheless, the existing clinical information about both of these compounds possibly makes such a combination an attractive therapeutic option for advanced hormone-refractory prostate cancer. It is also notable because DU-145 is particularly resistant to most retinoids, requiring high doses to achieve modest inhibition; however, significant inhibition was achieved in the current study with a relatively low dose of retinoid in combination with *Bcl-2* antisense oligonucleotide.

### ACKNOWLEDGEMENTS

This manuscript was supported by NIH grants CA43277, CA42710, CA70675-01 and CA26038 and also in part by the CaPCure and Concern Foundations and the Parker Hughes Trust. Dr H Phillip Koeffler is a member of the UCLA Jonsson Comprehensive Cancer Center and holds an endowed Mark Goodson Chair of Oncology Research at Cedars-Sinai Medical Center/UCLA School of Medicine.

### REFERENCES

Apakama I, Robinson MC, Walter NM, Charlton RG, Royds JA, Fuller CE, Neal DE and Hamdy FC (1996) Bcl-2 overexpression combined with p53 protein accumulation correlates with hormone-refractory prostate cancer. *Br J Cancer* **74**: 1258–1262

Barbareschi M, Caffo O, Veronese S, Leek RD, Fina P, Fox S, Bonzanini M, Girlando S, Morelli L, Eccher C, Pezzella F, Doglioni C, Dalla Palma P and Harris A (1996) Bcl-2 and p53 expression in node-negative breast carcinoma: a study with long-term follow-up. *Human Pathol* **27**: 1149–1155

Bauer JJ, Sesterhenn IA, Mostofi FK, Mcleod DG, Srivastava S and Moul JW (1996) Elevated levels of apoptosis regulatory proteins p53 and bcl-2 are independent prognostic biomarkers in surgically treated clinically localized prostate cancer. *J Urol* **156**: 1511–1516

Bercham GJ, Bosseler M, Sugars LY, Voeller HJ, Zeitlin S and Gelmann EP (1995) Androgens induce resistance to *bcl-2*-mediated apoptosis in LNCaP prostate cancer cells. *Cancer Res* **55**: 735–738

Blutt SE, Allegretto EA, Wesley Pike J and Wiegel NL (1997) 1,25-Dihydroxyvitamin $D_3$ and 9-*cis*-retinoic acid act synergistically to inhibit the growth of LNCaP prostate cells and cause accumulation of cell in $G_1$. *Endocrinology* **138**: 1491–1497

Campbell MJ, Park S, Uskokovic M, Dawson MI and Koeffler HP (1997) RAR expression plays a role in the clonal inhibition of prostate cancer cells mediated by combination of novel retinoids and a vitamin $D_3$ analog. *Endocrinology* (in press)

Campos L, Sabido O, Rouault JP and Guyotat D (1994) Effects of BCL-2 antisense oligonucleotides on in vitro proliferation and survival of normal marrow progenitors and leukemic cells. *Blood* **84**: 595–600

Carrol AG, Voeller HJ, Sugars L and Gelmann EP (1993) p53 oncogene mutations in three prostate cancer cell lines. *Prostate* **23**: 123–134

Chomienne C, Fenaux P and Degos L (1996) Retinoid differentiation therapy in promyelocytic leukemia. *Faseb J* **10**: 1025–1030

Dahiya R, Boyle B, Park H-D, Kurhanewicz J, Macdonald JM and Narayan P (1994*a*) 13-*cis*-retinoic acid-mediated growth inhibition of DU-145 human prostate cancer cells. *Biochem Mol Bio Internat* **32**: 1–12

Dahiya R, Park H-D, Cusick J, Vessela RL, Fournier G and Narayan P (1994*b*) Inhibition of tumorigenic potential and prostate-specific antigen expression in LNCaP human prostate cancer cell line by 13-*cis*-retinoic acid. *Int J Cancer* **59**: 126–132

Danesi R, Figg WD, Reed E and Myers CE (1995) Paclitaxel (taxol) inhibits protein isoprenylation and induces apoptosis in PC-3 human prostate cancer cells. *Mol Pharm* **47**: 1106–1111

Delia D, Aiello A, Formelli F, Fontanella E, Costa A, Miyashita T, Reed JC and Pierotti MA (1995) Regulation of apoptosis induced by the retinoid N-(4-hydroxyphenyl) retinamide and effect of deregulated Bcl-2. *Blood* **85**: 359–367

Gaddipati JP, Mcleod DG, Heidenberg HB, Sesterhenn IA, Finger MJ, Moul JW and Srivastava S (1994) Frequent detection of codon 877 mutation in the androgen receptor gene in advanced prostate cancers. *Cancer Res* **54**: 2861–2864

Golstein P (1997) Controlling cell death. *Science* **275**: 1081–1082

Higashiyama M, Doi O, Kodama K, Yokouchi H and Tateishi R (1996) Bcl-2 oncoprotein expression is increased especially in the portion of small cell carcinoma within the combined type of small-cell lung cancer. *Tumor Biol* **17**: 341–344

Hsieh TC, Xu W and Chiao JW (1995*a*) Growth regulation and cellular changes during differentiation of human prostatic cancer LNCaP cells as induced by T-lymphocyte-conditioned medium. *Exp Cell Res* **218**: 137–143

Hsieh TC, Ng C and Wu JM (1995*b*) The synthetic retinoid N-(4-hydroxyphenyl) retinamide (4-HPR) exerts antiproliferative and apoptosis-inducing effects in the androgen-independent human prostatic JCA-1 cells. *Biochem Mol Bio Intern* **37**: 499–506

Huang M, Ye Y, Chen SR, Chai JR, Lu JX, Zhao L, Gu LJ and Wang ZY (1988) Use of all-*trans* retinoic acid in the treatment of acute promyelocytic leukemia. *Blood* **72**: 567–572

Isaacs WB, Bova GS, Morton RA, Bussemakers MJ, Brooks JD and Ewing CM (1994) Genetic alterations in prostate cancer. *Cold Spring Harbor Symp Quant Biol* **59**: 653–659

Jacobsen SJ, Katusic SK, Bergstralh EJ, Oesterling JE, Ohrt DG, Klee G, Chute CG and Lieber MM (1995) Incidence of prostate cancer diagnosis in the eras before and after serum prostate-specific antigen testing. *JAMA* **274**: 1445–1449

Kantarjian HM, Estey EH and Keating MA (1996) New chemotherapeutic agents in acute myeloid leukemia. *Leukemia* **10**(suppl. 1): S4–6

Kazmi SM, Plante RK, Visconti V and Lau CY (1996) Comparison of retinoid N-(4-hydroxyphenyl) retinamide and all-trans-retinoic acid in the regulation of retinoid receptor-mediated gene expression in human breast cancer cell lines. *Cancer Res* **56**: 1056–1062

Keith FJ, Bradbury DA, Zhu YM and Russel NH (1995) Inhibition of bcl-2 with antisense oligonucleotides induces apoptosis and increases the sensitivity of AML blasts to Ara-C. *Leukemia* **9**: 131–138

Krajewska M, Krajewski S, Epstein JI, Shabaik A, Sauvageot J, Song K, Kitada S and Reed JC (1996) Immunohistochemical analysis of bcl-2, bax, bcl-X and mcl-1 expression in prostate cancers. *Am J Pathol* **148**: 1567–1576

Li CJ, Wang C and Pardee AB (1995) Induction of apoptosis by beta-lapachone in human prostate cancer cells. *Cancer Res* **55**: 3712–3715

Li X and Daryzynkiewicz Z (1995) Labelling DNA strand breaks with BrdUTP. Detection of apoptosis and cell proliferation. *Cell Prolif* **28**: 571–579

Liu L, Shack S, Stetler-Stevenson WG, Hudgins WR and Samid D (1994) Differentiation of cultured human melanoma cells induced by the aromatic fatty acids phenylacetate and phenylbutyrate. *J Invest Derm* **103**: 335–340

Lotan R (1994) Suppression of squamous cell carcinoma growth and differentiation by retinoids. *Cancer Res* **54**(suppl.) 1987–1990

McConkey DJ, Greene G and Pettaway CA (1996) Apoptosis resistance increases with metastatic potential in cells of the human LNCaP prostate carcinoma line. *Cancer Res* **56**: 5594–5599

Niles RM (1995) Use of vitamins A and D in chemoprevention and therapy of cancer: control of nuclear receptor expression and function. Vitamins, cancer and receptors. *Adv Exp Med Biol* **375**: 53–63

Novichenko N, Konno S, Nakajima Y, Hsieh TC, Xu W, Turo K, Ahmed T and Chiao JW (1995) Growth attenuation in a human prostate cell line mediated by a phorbolester. *Proc Soc Exp Biol Med* **209**: 152–156

Paquette RL and Koeffler HP (1992) Differentiation therapy. *Hem/Oncol Clin N Am* **6**: 687–706

Parker SL, Tong T, Bolden S and Wingo PA (1997) Cancer Statistics, 1997. *CA Cancer J Clin* **47**: 5–27

Peck R and Bollag W (1991) Potentiation of retinoid-induced differentiation of HL-60 and U937 cell lines by cytokines. *Eur J Cancer* **27**: 53–57

Pemrick SM, Lucas DA and Grippo JF (1994) The retinoid receptors. *Leukemia* **8** (suppl. 3): S1–S10

Pienta KJ, Nguyen NM and Lehr JE (1993) Treatment of prostate cancer in the rat with the synthetic retinoid fenretinamide. *Cancer Res* **53**: 224–226

Planchon SM, Wuerzberger S, Frydman B, Witiak DT, Hutson P, Church DR, Wilding G and Boothman A (1995) Beta-lapachone-mediated apoptosis in human promyelocytic leukemia (HL-60) and human prostate cancer cells: a p53-independent response. *Cancer Res* **55**: 3706–3711

Pollard M, Luckert PH, and Sporn MB (1991) Prevention of primary prostate cancer in Lobund-Wistar rats by N-(4-hydroxyphenyl) retinamide. *Cancer Res* **51**: 3610–3611.

Ponzoni M, Bocca P, Chiesa V, Decensi A, Pistoia V, Raffaghello L, Rozzo C and Montaldo PG (1995) Differential effects of retinoid N-(4-hydroxyphenyl) retinamide and retinoic acid on neuroblastoma cells: apoptosis versus differentiation. *Cancer Res* **55**: 833–861

Raffo AJ, Perlman H, Chen MW, Day ML, Streitman JS and Buttyan R (1995) Overexpression of bcl-2 protects prostate cancer cells from apoptosis in vitro

© Cancer Research Campaign 1998

and confers resistance to androgen depletion in vivo. *Cancer Res* **55**: 4438–4445

Rampino N, Yamamoto H, Ionov Y, Li Y, Sawai H, Reed JC and Perucho M (1997) Somatic frameshift mutations in the BAX gene in colon cancers of the microsatellite mutator phenotype. *Science* **275**: 967–969

Sachs L and Lotem J (1993) Control of programmed cell death in normal and leukemic cells: new implications for therapy. *Blood* **82**: 15–21

Samid D, Shack S and Myers CE (1993) Selective growth arrest and phenotypic reversion of prostate cancer cells in vitro by nontoxic pharmacological concentrations of phenylacetate. *J Clin Invest* **91**: 2288–2295

Saunders DE, Christensen C, Williams JR, Wappler NL, Lawrence WD, Malone JM, Malviya VK and Deppe G (1995) Inhibition of breast and ovarian carcinoma cell growth by 1,25-dihydroxyvitamin $D_3$ combined with retinoic acid or dexamethasone. *Anti-Cancer Drugs* **6**: 562–569

Smith MR, Abubakr Y, Mohammad R, Xie T, Hamden M and al-Katib A (1995) Antisense oligodeoxyribonucleotide down-regulation of bcl-2 gene expression inhibits growth of the low-grade non-Hodgkin's lymphoma cell line WSU-FSCCL. *Cancer Gene Ther* **2**: 207–212

Suzuki A, Matsuzawa A and Iguchi T (1994) Down regulation of Bcl-2 is the first step in Fas-mediated apoptosis of male reproduction tract. *Oncogene* **13**: 31–37

Takayama S, Sato T, Krajewski S, Kochel K, Irie S, Millan JA and Reed JC (1995) Cloning and functional analysis of BAG-1: a novel Bcl-2-binding protein with anti-cell death activity. *Cell* **80**: 279–284

Tamimi Y, Bringuier PP, Smit F, van Bokhoven A, Debruyne FM and Schalken JA (1996) p16 mutations/deletions are not frequent events in prostate cancer. *Br J Cancer* **74**: 120–122

Trump DL (1994) Retinoids in bladder, testis and prostate cancer: epidemiologic, pre-clinical and clinical observations. *Leukemia* **8**(suppl.): S50–S54

de Vos S, Dawson MI, Holden S, Le J, Wang A, Cho S, Chen D and Koeffler HP (1997) Effects of Retinoid X Receptor (RXR)-class selective ligands on prostate cancer cell proliferation. *Prostate* **32**(2): 115–121

Wang TT and Phang JM (1996) Effect of retinoid N-(4-hydroxyphenyl) retinamide on apoptosis in human breast cancer cells. *Cancer Lett* **107**: 65–71

Welsh J (1994) Induction of apoptosis in breast cancer cells in response to vitamin D and antiestrogens. *Biochem Cell Biol* **72**: 537–545

Wright AS, Thomas LN, Douglas RC, Lazier CB and Rittmaster RS (1996) Relative potency of testosterone and dihydroxytestosterone in preventing atrophy and apoptosis in the prostate of the castrated rat. *J Clin Invest* **98**: 2558–2563

Yang E and Korsmeyer SJ (1996) Molecular thanatopsis: a discourse on the BCL-2 family and cell death. *Blood* **2**: 386–401

Zhai S, Yaar M, Doyle SM and Gilchrist BA (1996) Nerve growth factor rescues pigment cells from ultraviolet-induced apoptosis by upregulating BCL-2 levels. *Exp Cell Res* **224**: 335–343

Zhang W, Grasso L, McClain CD, Gambel AM, Cha Y, Travali S, Deisseroth AN and Mercer WE (1995) p53-independent induction of WAF1/CIP1 in human leukaemia cells is correlated with growth arrest accompanying monocyte/macrophage differentiation. *Cancer Res* **55**: 668–674

*© Cancer Research Campaign 1998*